# Dynamic Metasurface Aperture as Smart Around-the-Corner Motion Detector

**DOI:** 10.1038/s41598-018-24681-9

**Published:** 2018-04-25

**Authors:** Philipp del Hougne, Mohammadreza F. Imani, Timothy Sleasman, Jonah N. Gollub, Mathias Fink, Geoffroy Lerosey, David R. Smith

**Affiliations:** 1 Institut Langevin, CNRS UMR 7587, ESPCI Paris, PSL Research University, 1 rue Jussieu, 75005 Paris, France; 2 Center for Metamaterials and Integrated Plasmonics, Duke University, Department of Electrical and Computer Engineering, Durham, North Carolina 27708 USA

## Abstract

Detecting and analysing motion is a key feature of *Smart Homes* and the connected sensor vision they embrace. At present, most motion sensors operate in line-of-sight Doppler shift schemes. Here, we propose an alternative approach suitable for indoor environments, which effectively constitute disordered cavities for radio frequency (RF) waves; we exploit the fundamental sensitivity of modes of such cavities to perturbations, caused here by moving objects. We establish experimentally three key features of our proposed system: (i) ability to capture the temporal variations of motion and discern information such as periodicity (“smart”), (ii) non line-of-sight motion detection, and (iii) single-frequency operation. Moreover, we explain theoretically and demonstrate experimentally that the use of dynamic metasurface apertures can substantially enhance the performance of RF motion detection. Potential applications include accurately detecting human presence and monitoring inhabitants’ vital signs.

## Introduction

With the advent of futuristic concepts such as *Smart Homes* and the *Internet of Things*^1^, there is an increasing need for sensors that can detect motion in complicated indoor environments and also extract useful information about the motion^2^. Furnished indoor environments usually have complicated, irregular geometries^3–6^ such that typical Doppler shift based motion detectors are quickly overstrained, in particular by motion outside their line-of-sight; moreover, they are easily fooled by fans and similar rotating objects. An alternative emerging approach is the use of existing ambient signals such as Wi-Fi to monitor the environment. While tremendous strides have been made towards this goal^7–11^, the use of such ambient waves usually requires complicated signal and statistical processing techniques. Furthermore, reliance on Wi-Fi signals prevents this approach from becoming ubiquitous. Accurate human presence detection is important for smart buildings, for instance to identify intruders as well as to reliably switch on or off lights and heating. The latter may enable residential homes to save around a third of their energy consumption^12^. Furthermore, intelligent motion detectors can be envisioned to monitor vital signs, such as respiration rate, to improve the support and monitoring of infants and the elderly.

We address the above challenges with a motion detector that leverages the complexity of indoor environments rather than attempting to disentangle it. Indoor radio frequency (RF) wave propagation is dominated by complex multi-path trajectories due to reflections off countless irregularly placed and shaped objects that can range from metallic cupboards to steel girders. Radiowaves become trapped in these environments and establish speckle-like stationary wave fields, such that from a physics perspective we are dealing with a (low quality factor) disordered cavity. In light of this interpretation of waves trapped in indoor environments, recent works have suggested the idea of changing the disordered cavity’s geometry, for example to boost the received signal level for indoor wireless communication^13–15^ or for RF harvesting^16,17^. Rather than physically changing the geometry, simple reflectarrays partially covering the room’s walls can enable one to reconfigure the room’s boundary conditions to obtain the desired focused wave fields. This concept of wavefront shaping, originally developed in optics^18^, has recently been transposed to the microwave domain^19,20^.

The underlying principle of our motion detector is the sensitivity of an indoor environment’s electromagnetic response to small perturbations – just as a cavity’s modes are extremely sensitive to its geometry. Even small perturbations of a room’s or cavity’s geometry notably modify its mode structure. The extraordinary sensitivity of dynamic light scattering to displacements as low as *λ*/100 in multiple scattering media is well-known from diffusing-wave spectroscopy^21^ and easily transposed to our system by applying the theory of images to the cavity interfaces^22^. All objects inside a cavity are of course part of the geometry taken into account by the wave equation that defines the cavity modes; thus any motion of objects inside a cavity constitutes a perturbation of the cavity modes. The latter is true for any object that interacts with the wave field, be it through scattering or absorption^23,24^. A convenient way to probe the wave field inside a cavity is to measure the transmitted field *S*(*f*) between two antennas inside the cavity. Continuous measurements tend to reveal any motion of objects inside the cavity, even outside the line-of-sight of the two probe antennas, since the waves explore the entire cavity to establish the stationary wave fields. Moreover, any temporal variations should leave signatures on the repeatedly measured transmissions, from which useful information about the motion can be extracted. For example, a periodically moving object such as a fan can be distinguished from the periodic fluctuations of the received signal.

The starting point for understanding the physics of a transmission measurement between two probe antennas in an electromagnetic cavity is the dyadic Green’s function $\overline{\overline{G}}(\mathop{{r}_{t}}\limits^{\longrightarrow},\mathop{{r}_{r}}\limits^{\longrightarrow},f)$ between two locations $\mathop{{r}_{t}}\limits^{\longrightarrow}$ and $\overrightarrow{{r}_{r}}$ inside the cavity, defined as

1 $$\overline{\overline{G}}(\mathop{{r}_{t}}\limits^{\longrightarrow},\mathop{{r}_{r}}\limits^{\longrightarrow},f)=\sum _{n=1}^{\infty }\,\frac{\mathop{{\psi }_{n}}\limits^{\longrightarrow}(\mathop{{r}_{t}}\limits^{\longrightarrow})\otimes \mathop{{\psi }_{n}}\limits^{\longrightarrow}({\vec{r}}_{r})}{\frac{2\pi }{c}({f}_{n}^{2}-{f}^{2})}=\sum _{n=1}^{\infty }\,\overline{\overline{{\varphi }_{n}}}(\mathop{{r}_{t}}\limits^{\longrightarrow},\mathop{{r}_{r}}\limits^{\longrightarrow},f),$$

where $\mathop{{\psi }_{n}}\limits^{\longrightarrow}$ are the real eigenfunctions of the closed system, *f*_*n*_ the corresponding eigenfrequencies and $\otimes$ denotes the tensor product operation^25–27^. This description can account for homogeneous (global) losses by including them in *f*_*n*_ which then becomes complex^28^. At a given working frequency *f*_0_, the Green’s function $\overline{\overline{G}}(\mathop{{r}_{t}}\limits^{\longrightarrow},\mathop{{r}_{r}}\limits^{\longrightarrow},{f}_{0})$ can thus be expressed as sum of the *N* cavity modes that overlap at *f*_0_ due to the finite line-widths of the modes, originating from losses and leakages. We label the set of the modes contributing at the working frequency as ${\rm{\Phi }}=\{\overline{\overline{{\varphi }_{{n}_{1}}}},\,\overline{\overline{{\varphi }_{{n}_{2}}}},\,\ldots \,\overline{\overline{{\varphi }_{{n}_{N}}}}\}$, and can estimate *N* with Weyl’s Law^19,29,30^,

2 $$N\approx \frac{4V\pi {f}_{0}^{3}}{{c}^{3}Q},$$

*V* and *Q* being the cavity’s volume and quality factor, respectively.

In principle, all the previously discussed information about motion-induced mode perturbations should be encoded in the Green’s function, even if it is known only at a single frequency. Yet in reality, we do not measure $\overline{\overline{G}}(\mathop{{r}_{t}}\limits^{\longrightarrow},\mathop{{r}_{r}}\limits^{\longrightarrow},{f}_{0})$ directly. The different contributions to the modal sum are weighted depending on how well the probe antennas, whose centers are located at $\mathop{{r}_{t}}\limits^{\longrightarrow}$ and $\mathop{{r}_{r}}\limits^{\longrightarrow}$, are able to excite the corresponding cavity modes. The total signal *S* transmitted from the port of the transmitter to the port of the receiver can hence be modeled as

3 $$S(\mathop{{r}_{t}}\limits^{\longrightarrow},\mathop{{r}_{r}}\limits^{\longrightarrow},{f}_{0})\propto \sum _{n={n}_{1}}^{{n}_{N}}\,{\int }_{{A}_{t,i}}\,{\int }_{{A}_{r,j}}\,\mathop{{E}_{t}}\limits^{\longrightarrow}(\mathop{{{r}}_{t,i}}\limits^{\longrightarrow},{f}_{0})\overline{\overline{{\varphi }_{n}}}(\mathop{{{r}}_{t,i}}\limits^{\longrightarrow},\mathop{{{r}}_{r,j}}\limits^{\longrightarrow},{f}_{0}){\mathop{{E}_{r}}\limits^{\longrightarrow}}^{T}(\mathop{{{r}}_{r,j}}\limits^{\longrightarrow},{f}_{0})\,{\rm{d}}\mathop{{{r}}_{t,i}}\limits^{\longrightarrow}{\rm{d}}\mathop{{{r}}_{r,j}}\limits^{\longrightarrow},$$

where the superscript ^*T*^ denotes the transpose operation, *A*_*t*,*i*_ and *A*_*r*,*j*_ are the apertures and $\mathop{{{r}}_{t,i}}\limits^{\longrightarrow}$ and $\mathop{{{r}}_{r,j}}\limits^{\longrightarrow}$ the position along the apertures of the transmitting and receiving antenna, respectively. Since the probes can generally be extended antennas, integration with respect to $\mathop{{{r}}_{t,i}}\limits^{\longrightarrow}$ and $\mathop{{{r}}_{r,j}}\limits^{\longrightarrow}$ ensures taking into account the transmission from across the entire transmit aperture to the entire receive aperture (port to port); the variables *A*_*t*,*i*_ and $\mathop{{{r}}_{t,i}}\limits^{\longrightarrow}$, as well as *A*_*r*,*j*_ and $\mathop{{{r}}_{r,j}}\limits^{\longrightarrow}$, are implicitly functions of $\mathop{{r}_{t}}\limits^{\longrightarrow}$, and $\mathop{{r}_{r}}\limits^{\longrightarrow}$, respectively. Intuitively, Eq. 3 may be interpreted as a double overlap integral that evaluates for each polarization how closely the radiation patterns of the transmitter $\mathop{{E}_{t}}\limits^{\longrightarrow}$ and receiver $\mathop{{E}_{r}}\limits^{\longrightarrow}$ at the respective antenna apertures match each of the *N* relevant speckle-like modal contributions $\overline{\overline{{\varphi }_{n}}}$ to the Green’s function. Exact expressions for the coupling efficiencies have been derived for specific cases, see refs^31–33^. Note that neither Φ nor $\mathop{{E}_{t}}\limits^{\longrightarrow}$ and $\mathop{{E}_{r}}\limits^{\longrightarrow}$ are explicitly known, nor sought to be estimated here; the only quantity we measure is *S*. Nonetheless, understanding the physics behind this simple measurement is crucial to interpreting why and how our proposed system works.

A motion detector, unlike an imaging device, only seeks to establish the occurrence of motion, and possibly discern some of its characteristics. Motion of objects inside the cavity will perturb the cavity modes, causing changes to Φ as well as to the coupling of transmitting and receiving antennas to the cavity, since the cavity modes’ spatial patterns are modified. Ultimately, by measuring *S* we are monitoring a weighted sum of cavity modes that changes in response to any motion inside the cavity. Yet, small perturbations might cause only small changes to some contributions of the sum in Eq. 3 that cancel one another or fall below the noise floor.

The above observations point towards the need for probing the wave field and exciting its constituting modes in several independent ways. Two trivial options come to mind. First, adding more antenna pairs at independent locations where the local patterns of the cavity modes are different, using the dependence of $\overline{\overline{{\varphi }_{n}}}(\mathop{{r}_{t}}\limits^{\longrightarrow},\mathop{{r}_{r}}\limits^{\longrightarrow},{f}_{0})$ on $\mathop{{r}_{t}}\limits^{\longrightarrow}$ and $\mathop{{r}_{r}}\limits^{\longrightarrow}$. This option, however, quickly becomes invasive for a sensing application and cumbersome to implement, having to connect and switch between different antenna pairs. Secondly, since the cavity modes’ patterns are speckle-like not only in space but also in the frequency domain, one might consider working with a single pair of probe antennas and measuring at several independent frequencies that are separated by at least a correlation frequency Δ*f*_*corr*_ = *f*_0_/*Q*. Each independent frequency would yield independent $\overline{\overline{{\varphi }_{n}}}(\mathop{{r}_{t}}\limits^{\longrightarrow},\mathop{{r}_{r}}\limits^{\longrightarrow},{f}_{0})$, due to the dependence on *f*_0_, and the local mode patterns at the apertures of the receiving and transmitting antenna would be different for each independent working frequency *f*_0_. However, this takes away the advantages of single-frequency operation, namely not having to design complex and costly broadband hardware as well as minimising any spectrum allotment problems.

**Figure 1:**
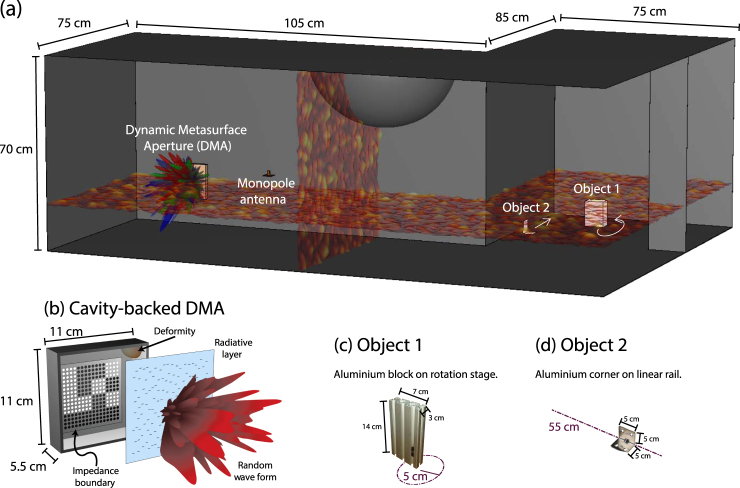
(**a**) Schematic of experimental set-up. The transmission between a Dynamic Metasurface Aperture (DMA) and a monopole antenna is measured in a (large) disordered metallic cavity. The wavelength of operation is 1.55 cm. The cavity wave field is speckle-like as visualised by the vertical and horizontal slices. Two objects, outside the line-of-sight of the antenna pair, can be moved. (**b**) Schematic of the cavity-backed DMA: a (small) open, disordered cavity with partially tunable boundary conditions. [Figure adapted from ref.^36^]. (**c**) Object 1 is a small aluminium block mounted onto a rotation stage. (**d**) Object 2 is an L-shaped aluminium corner mounted onto a linear rail.

These concerns motivate the use of a dynamic metasurface aperture (DMA) as at least one of the probe antennas. DMAs are devices capable of producing arbitrary radiation patterns that can be varied with simple electronic controls. They have become an attractive tool for RF imaging^34^ because they considerably simplify the imaging system’s physical layer in the context of computational imaging schemes, shifting the burden from hardware to software. A variety of DMA designs exists for both beam forming and imaging applications, ranging from one-dimensional microstrip implementations to disordered three-dimensional cavities^35–37^. Here, we use the latter, which enables wavefront shaping. The DMA we employ, the same as that in ref.^36^, is an electrically large (but much smaller than an indoor room) disordered cavity with partially controllable boundary conditions, as illustrated in Fig. 1(b). The controllable boundary is created by placing an artificial impedance surface on one wall and enabling binary tunability with varactor diodes. This cavity is excited by a simple rectangular waveguide and the wall opposite the tunable impedance surface is perforated so that it radiates, producing complex field patterns that depend on the DMA’s boundary configurations. Used as a transmitting antenna in different random configurations of its controllable boundary, the DMA will emit arbitrary radiation patterns $\mathop{{E}_{t}}\limits^{\longrightarrow}$ that each overlap differently with the cavity modes’ spatial patterns in Eq. 3. In essence, to have different probes of the cavity modes, rather than exploring different $\overline{\overline{{\varphi }_{n}}}$, the DMA conveniently enables one to alter $\mathop{{E}_{t}}\limits^{\longrightarrow}$, at fixed $\overline{\overline{{\varphi }_{n}}}$.

DMAs can be implemented with a simple architecture and draw only small amounts of power, making them an attractive option to overcome the aforementioned concerns. A naive alternative to using a DMA to achieve diverse radiation patterns—using many probe antennas and applying different random weights to them before integrating them—demonstrates how advantageous the DMA’s electrically-large size and multiplexing nature are. Furthermore, DMAs offer the ability to tune their radiation pattern at rapid rates (on the order of a few MHz), leading to the unique opportunity to sample the scene and acquire its temporal variations. Invoking learning algorithms such as recurrent neural networks^38,39^, temporal variations of the scene may be analysed to discern the source of motion (e.g. a human or a fan). In another instance, the temporal variation can be used to monitor life signs, e.g. via examining a subject’s respiration rate. (We refer readers interested in other applications of metadevices to refs^40–43^).

In this work, we present a proof-of-concept experiment of a motion detector based on dynamic metasurface apertures. First, we introduce the experimental set-up and general data processing procedure for motion detection. We go on to introduce false positive and negative rates as a measure of motion detection accuracy. We evaluate these rates as a function of the number of measurements with random DMA configurations used to probe the room. Moreover, we quantify by how much the performance in detecting dynamic scatterers in an otherwise static disordered environment can be improved by using a DMA compared to non-tunable antennas. Finally, we show that we can precisely identify intervals in which the detected motion was purely periodic and even estimate its period accurately, suggesting the possibility to acquire temporal signatures of the motion within the room.

## Results

### Experimental Setup

For this proof-of-concept demonstration, we choose to work in a disordered metallic cavity which provides a well-controlled environment for our tests. The cavity is very large (1.4 m^3^) compared to the wavelength of 1.55 cm at our working frequency of *f*_0_ = 19.4 GHz. Operating in the K-band ensures sensitivity to motion on the order of the millimeter while maintaining a reasonably small DMA size of a few centimeters. We have deliberately chosen an L-shape cavity layout, as shown in Fig. 1(a), to ensure that we can create motion that is clearly outside the line-of-sight of the probing antennas—a DMA and a monopole antenna for transmitter and receiver, respectively. We estimate the cavity’s quality factor from the decay rate of the transmission spectrum’s inverse Fourier transform to be about 2000 such that by Eq. 2 there are about 2400 modes overlapping at the working frequency. Note that this is not a contrived experimental setup but in fact very similar to a strong room or vault (such as that found in a bank), for instance, where motion detection is crucial and yet difficult due to the complicated geometry.

As depicted in Fig. 1, two objects are placed “around-the-corner”. Object 1 is a small aluminium block that can be moved on a rotation stage in a circle of radius 5 cm. Object 2 is an aluminium corner mounted onto a linear 55 cm rail. We deliberately work with metallic objects to mimic the human skin’s high reflectivity characteristics in the K-band^44,45^. The dimensions of both objects (cf. Fig. 1) are only a few wavelengths and are tiny compared to the cavity size. To some extent, our setup resembles the use of mode-stirrers^46^ in reverberation chambers to achieve maximal mode perturbations for electromagnetic compatibility analysis; in contrast, we try to detect minimal perturbations here.

We use the cavity-backed DMA described in detail in ref.^36^ to test the proposed concepts. This DMA, illustrated in Fig. 1(b), is an electrically large disordered cavity (11 × 11 × 5.5 cm^3^) with 150 randomly-placed irises on one of its walls, such that the multitude of distinct modes supported by the cavity couple to spatially distinct radiative modes. Another DMA cavity wall is covered with a 4 × 4 matrix of binary tunable impedance pixels (each of which contains 4 × 4 elements with a parallel voltage bias), enabling one to independently change the reflection phase of each pixel by *π*^47^. This tuning is facilitated by MACOM MAVR-011020-1411 varactor diodes which provide continuous capacitive tuning, but we use a binary 0 V/5 V scheme to maintain a simple system. By tuning this voltage with an Arduino microcontroller, the modes supported by the cavity and consequently the radiated fields can be tuned. It is worth noting that an ultimate real-life implementation may prefer alternative DMA designs such as the planer devices employed in refs^35,48,49^.

In our experiment we work on an arbitrary time scale; we take a series of transmission measurements with a network analyser at instant *t*_*i*_, then Object 1 or both objects or neither move “instantaneously”, and then we take the next series of transmission measurements at instant *t*_*i*+1_. This enables well-controlled experiments and is a good approximation to future real-life applications, where improved electronics and a custom^45,50^ single-frequency radio could easily enable the transmission measurements to be carried out at MHz frame rates (relative to which any human or mechanical motion is almost static for the duration of a few hundred measurements).

**Figure 2:**
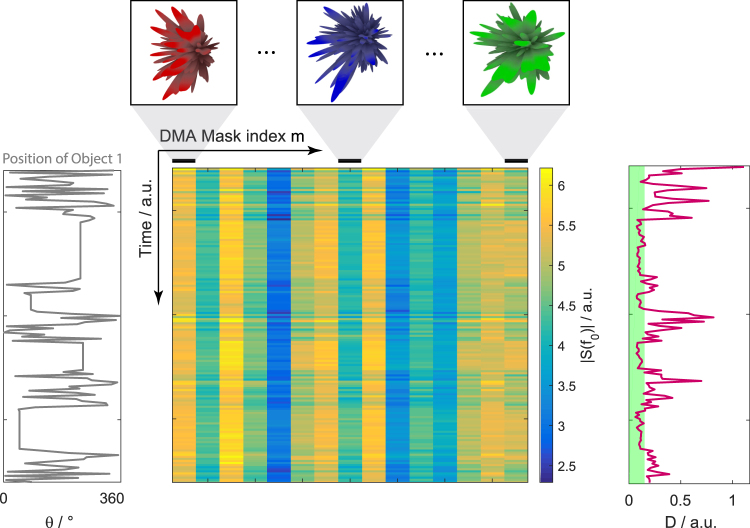
We measure a complex-valued matrix *W* whose entry *W*(*t*_*i*_, *m*) is the transmission *S*(*f*_0_) between the DMA and the monopole antenna at time *t*_*i*_ for the *m*th random DMA configuration. Here we display |*W*| with time along the vertical and the DMA mask index along the horizontal axis. The angular position of the moving Object 1 as a function of time is indicated, as well as some sample radiation patterns for different random DMA configurations. On the right we show how thresholding the quantity *D* calculated from *W* according to Eq. 4 identifies when motion occurred and when not.

Over the course of the experiment, we obtain a complex-valued matrix *W*, as indicated in Fig. 2, containing for each moment in time the transmission values at the working frequency for a set of random DMA configurations. The entry *W*(*t*_*i*_, *m*) is the transmission *S*(*f*_0_) between the DMA and the monopole antenna at time *t*_*i*_ for the *m*th random DMA configuration. To decide whether motion occurred between instants *t*_*i*_ and *t*_*i*+1_, we first estimate by how much the transmission changed:

4 $$D={\langle |W({t}_{i+1},m)-W({t}_{i},m)|\rangle }_{m},$$

where 〈…〉_*m*_ denotes averaging over the measurements taken at each time step. Note, as discussed in the following section, that this averaging has a deeper statistical motivation beyond simply improving the signal-to-noise ratio. We then need to compare the value of *D* to a threshold *D*_*thresh*_ related to the noise floor. We estimate *D*_*thresh*_ as the average of *D* plus three times its standard deviation over 100 “initiation” measurements known to be motionless. As illustrated in Fig. 2, by inspection the binary decision of whether *D* lies above or below the threshold reliably correlates with the occurrence of motion.

### The benefit of using a Dynamic Metasurface Aperture

Next, we evaluate more precisely how well the “around-the-corner” motion detector works and we investigate its dependence on the number of measurements *M* at every moment in time. To quantify the performance, we estimate the false positive and false negative detection rates obtained for a long motion sequence of Object 1, including intervals of random motion and intervals of rest, similar to the example interval in Fig. 2. We define the false positive [negative] rate as the number of time steps at which motion was incorrectly detected to have occurred [not have occurred], compared to the total number of time steps. False negatives constitute a huge nuisance to the user, be it a *Smart Home*’s temperature and lighting control or an intruder alarm, and should be kept as low as possible. To demonstrate the benefit of using a DMA with *M* different radiation patterns $\mathop{{E}_{t}}\limits^{\longrightarrow}$ over making *M* repeated measurements with a non-tunable antenna of fixed radiation pattern $\mathop{{E}_{t}}\limits^{\longrightarrow}$, we conduct our measurements for three different scenarios. In the first case, we use *M* different DMA radiation patterns. In the second case, we use one fixed (randomly selected) DMA pattern and repeat the measurement *M* times (to have similar benefits of multiple measurements on the signal-to-noise ratio). In the third case, we replace the DMA with a standard K-band open-ended rectangular waveguide and repeat the measurement *M* times. It is worth noting that the latter cases resemble the technology used, for example, in motion detection based on ambient Wi-Fi fields, where a fixed antenna emits the signal.

**Figure 3:**
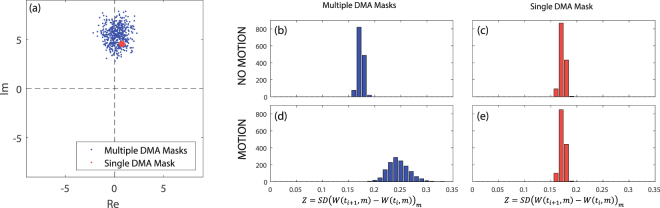
Separating the DMA’s effect from noise. (**a**) Representation in the complex plane of 500 transmission measurements for a given cavity, using 500 different random masks (blue) or 500 times the same random mask (red). (**b**–**e**) Histograms of *Z* over many realizations; *Z* is the standard deviation of the change between two consecutive series of transmission measurements, *Z* = *SD*(*W*(*t*_*i*+1_, *m*) − *W*(*t*_*i*_, *m*))_*m*_, as defined in Eq. 5. The top [bottom] row corresponds to cases in which no motion [motion] occurred in between the two measurement series.

To begin with, we visualise the extent to which the DMA can impact the transmission. In Fig. 3(a) we plot the measured *S*(*f*_0_) for 500 random DMA configurations in the same static cavity without any motion. Each blue dot is the sum of all the modes, weighted according to the corresponding DMA configuration, as expressed in Eq. 3. Recall that neither the modes Φ nor the radiation patterns $\mathop{{E}_{t}}\limits^{\longrightarrow}$ and $\mathop{{E}_{r}}\limits^{\longrightarrow}$ are explicitly known, but we observe the effect of their interplay in Fig. 3(a), as $\mathop{{E}_{t}}\limits^{\longrightarrow}$ is altered with the DMA. To unequivocally separate the DMA’s effect from simple measurement noise, we additionally plot for reference in red 500 measurements of a fixed, randomly selected DMA configuration, implying a fixed $\mathop{{E}_{t}}\limits^{\longrightarrow}$. Clearly the radius of the red cloud is very small compared to the blue one, implying that the observed transmission variations visualised with the blue dots are indeed due to the aforementioned mechanism of assigning different weights to different modes.

**Figure 4:**
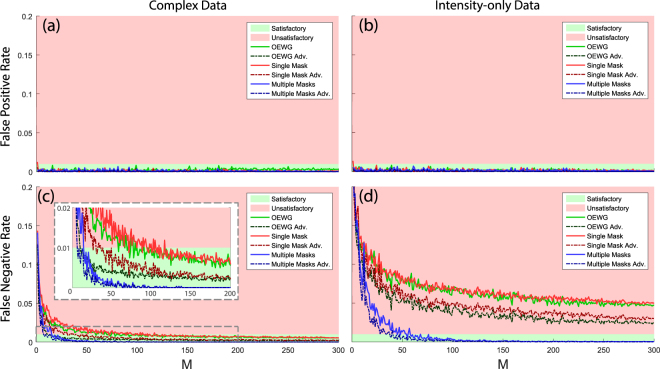
Dependence of False Positive [top row] and False Negative [bottom row] Rates on the number of measurements *M*, with [left] or without [right] phase information. We contrast using *M* different random DMA configurations [blue] with *M* repeated measurements of a fixed random DMA configuration [red] or a simple open ended waveguide probe (OEWG) [green]. The dash-dotted lines are obtained by using the knowledge that in a given interval motion does not occur or stop only at a single moment in time, cf. main text.

How useful will this be for motion detection? Our proposed approach relies on changes of the transmission as motion induces cavity geometry changes. The idea of using a DMA is that, on average, thanks to its ability to apply different weights to different modes, it should achieve a higher sensitivity than a non-tunable antenna. The idea of “on average” is crucial here. In a specific case, a non-tunable antenna could perform better or worse depending on the exact geometry; real-life scenarios are naturally never restricted to a single specific case since the geometry of an indoor environment constantly evolves as objects move. It is thus instructive to visualise the distribution of

5 $$Z=SD{(W({t}_{i+1},m)-W({t}_{i},m))}_{m}$$

over many realizations. This quantity *Z*, the standard deviation of the *M* transmission measurement changes between two consecutive time steps, is closely linked to the detection sensitivity. For a non-tunable antenna, *Z* = 0 in a noiseless system both if motion occurred or not, as confirmed by our measurements: with noise, the same narrow distribution is observed in Fig. 3(c,e). When motion occurs, in some DMA configurations the change of the transmission will have been large, enabling an easy detection, but in others minimal such that the motion was not detected. In contrast, with a dynamic antenna, the *Z* distribution for the case that motion occurred, cf. Fig. 3(d), is very different. When motion occurs, some of the random configurations will yield very large and others very small transmission changes for any given case, as revealed in Fig. 3(d). Thus the combination of many dynamic configurations will statistically, i.e. over a large number of realizations, outperform the non-tunable antenna. For any given scenario, sufficient measurements with different random DMA configurations will naturally always contain some configurations that yield large transmission changes, such that a zero false negative rate is achieved in Fig. 4(c). In contrast, a non-tunable antenna, employed for instance in sensors that utilise Wi-Fi signals, will inevitably come across scenarios in which its fixed radiation pattern corresponds only to a very small transmission change, explaining why the two approaches with fixed radiation pattern do not achieve a zero false neative rate in Fig. 4(c). The DMA proposed here is an ideal match to the physics of the problem: it can significantly reduce false negatives, while, as we show later, it simplifies the analysis to discern more information.

As a means of achieving a simplified hardware implementation, we explore the case of intensity-only measurements. While the loss of phase information does not prevent the DMA-based detection from reaching a zero false negative rate, at the cost of a few more measurements *M*, Fig. 4(d) clearly shows that the antennas with fixed radiation pattern have an unacceptably high false negative rate even with a vastly larger number of measurements *M*. The advantage of using a dynamic antenna is thus enhanced in the case of intensity-only measurements. Phaseless measurements make it impossible to pick up the difference between *S* and *S* + *δS* if |*S*| ≈ |*S* + *δS*|: this happens for instance if arg(*δS*) is close to arg(*S*) + *π*/2 as both quantities have the same amplitude, lying on the same circle around the origin of the complex plane. With an antenna of fixed radiation pattern, over a long series of measurements, this will inevitably occur a considerable amount of times, as evidenced by the pronounced difference between the results obtained with tunable and non-tunable antennas in Fig. 4(d). With a dynamic radiation pattern, among the tested random patterns there will also always be a few for which the motion of an object has mainly changed arg(*S*) without appreciable impact on |*S*|, but the majority of the random patterns will correspond to a detectable change of |*S*| — Fig. 3(a) clearly shows how random DMA configurations explore a wide variety of arg(*S*).

The motion detection performance can be augmented through the use of prior knowledge. Due to the envisaged MHz frame rates, if motion is [not] detected at a given instant *t*_*i*_, we assume this can only be the case if motion was also [not] detected at *t*_*i*−1_ or *t*_*i*+1_. This extra constraint is fitting in many applications such as, for example, considering that no intruder appears inside an indoor environment for only a few milliseconds. The results with this added information are traced in Fig. 4 as dash-dotted lines; for the false negative rate they outperform the results without this knowledge in all cases. The results of Fig. 4 demonstrate that adding some (albeit trivial) knowledge can enable one to achieve similar results with fewer measurements *M*. As a numerical comparison, if intensity-only data from multiple DMA configurations is used, an acceptable false negative rate is already reached with roughly *M* = 30 rather than 50, cf. Fig. 4(d).

**Figure 5:**
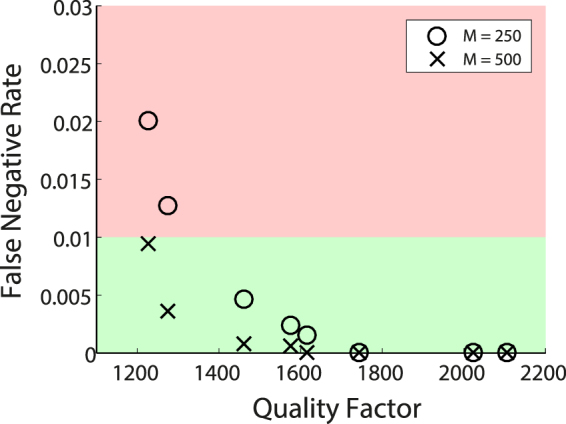
Impact of the room quality factor on the detection sensitivity, exemplified by the case of the false negative rate obtained with complex data and additional knowledge, using *M* = 250 and *M* = 500 random DMA masks.

Finally, we investigate the role of the room’s quality factor *Q* on the detection sensitivity. While our setup closely resembles metallic strong rooms, other environments such as residential and office rooms or warehouses are lossier and leakier for radiowaves. They thus have a lower quality factor. To lower our cavity’s quality factor, we place pieces of electromagnetic absorbers at random positions in our cavity. We repeated the previous experiment several times with different amounts of absorbing material. In Fig. 5 we show the detection sensitivity’s dependence on *Q*, exemplified by the case of the false negative rate using complex data from *M* random DMA configurations and the aforementioned additional knowledge. As expected, a higher *Q* factor, implying more reverberation as well as a higher signal-to-noise ratio, due to less absorption, gives better results. It becomes clear in Fig. 5 that as *Q* is decreased, more measurements *M* are needed to achieve the same detection sensitivity. Yet the gain in information content as function of the number of configurations used saturates somewhere between 150 and 200, due to correlations between the radiation patterns, as shown in ref.^36^. Needing significantly more than 200 DMA configurations at lower *Q* factors can hence be attributed to the deteriorated signal-to-noise ratio and does not seem to be an intrinsic sensitivity limitation, as seen for instance in Fig. 4(d) for intensity-only data from non-tunable antennas. The adverse effects of lower *Q*, in particular the lower signal-to-noise ratio, can thus be counterbalanced with simple measures such as increasing the receiver’s dynamic range as well as taking more measurements. It is worth emphasising that the DMA used in this experiment is not optimized for a high radiation efficiency or number of modes. In an ultimate implementation, such consideration can be taken into account to obtain the desired performance at a reasonable measurement speed. A further possibility is to use another DMA as the receiver, which significantly increases the number of independent measurements by providing the ability to tune both $\mathop{{E}_{t}}\limits^{\longrightarrow}$ and $\mathop{{E}_{r}}\limits^{\longrightarrow}$ (at the potential cost of a reduced signal-to-noise ratio). These trade-offs are beyond the scope of this article and are left for future works.

### Retrieving temporal signatures

**Figure 6:**
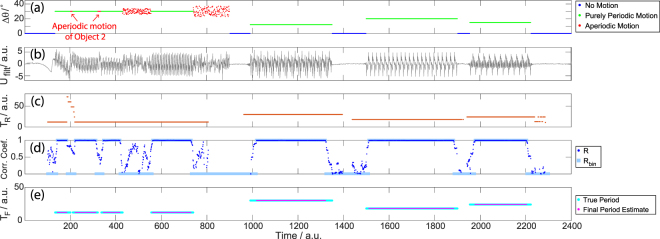
Detecting purely periodic motion and estimating its period, based on intensity-only data from *M* = 150 random DMA masks. (**a**) Angular displacement of Object 1 over a test interval, color-coded as shown in the legend. Aperiodicity is introduced once through random motion of Object 2 while Object 1 moves periodically and once by adding noise to the exact positions of Object 1 required for periodic motion (cf. Fig. 1). (**b**) The bandpass filtered quantity *U*_*filt*_, processed from the measurement matrix *W* as discussed in the main text (cf. Eq. 6), used to extract the desired information. (**c**) The raw estimate *T*_*R*_(*t*) of the motion’s periodicity. (**d**) The linear correlation coefficient *R* described in the main text, that confirms purely periodic motion detected in (**c**) if it is very close to unity. (**e**) The combined overall results, showing excellent agreement both in terms of distinguishing pure periodicity from no motion or aperiodicity as well as estimating the period.

Having explored the impact of several key parameters (number of measurements, phase information, *a priori* knowledge, room quality factor), we now move on to demonstrate the possibility of acquiring temporal signatures of the (non line-of-sight) motion. This is particularly useful to characterise and identify the source of motion. To demonstrate this capability, we focus on identifying purely periodic motion and the extraction of the corresponding period. This is of interest, for instance, to distinguish a rotating fan’s motion from that of an intruder or to monitor a patient’s respiration rate. With our setup we emulate a process, cf. Fig. 6(a), consisting of intervals with no motion, intervals with purely periodic motion of Object 1 for various periods, and intervals of aperiodic motion of Objects 1 and/or 2. The final case is introduced in one of two ways: once with random motion of Object 2 along its rail while Object 1 moves periodically, and once with random values added to the exact positions of Object 1 required for periodic motion. The results presented in the following are obtained from an intensity-only data set for *M* = 150 random DMA configurations.

The periodicity of the motion is encoded in repeating patterns in the transmission measurements. From the *M* measurements at each time step, we obtain the quantity

6 $$U(t)={\langle |W(t,m)|\rangle }_{m}.$$

Note that once again the averaging here is not solely a complicated way of improving the signal-to-noise ratio – the use of different masks is crucial to achieve an optimal sensitivity which is important for an accurate identification of purely periodic motion, too. We apply a bandpass filter, in our case prohibiting periods smaller than 2 or larger than 150, which is necessary due to measurement noise. The resultant filtered quantity *U*_*filt*_, displayed in Fig. 6(b), by inspection clearly contains periodic variation where expected.

To identify intervals of purely periodic motion based on *U*_*filt*_ and moreover estimate the period, we proceed in two steps. First, we apply a simple periodicity estimation^51^ through a sliding window of length Δ*t*_1_ = 200. For each window, it extracts the peaks of the autocorrelation and evaluates their separation. This is our raw period estimate *T*_*R*_ for the moment *t*_*i*_ that corresponds to the center of the respective window. As displayed in Fig. 6(c), this procedure accurately estimates the period and also identifies intervals without purely periodic motion — however, this method is not very accurate on short time scales and will return an incorrect estimate, for instance, when the periodic motion is distorted briefly.

The inaccuracy on short time scales motivates the second step of our processing, which seeks to validate the raw results on a time scale much shorter than Δ*t*_1_. Now we work with a sliding window of variable length. For every moment in time *t*_*i*_, we compare the signal in the intervals *t*_*i*_ − *T*_*R*_(*t*_*i*_) < *t* < *t*_*i*_ and *t*_*i*_ < *t* < *t*_*i*_ + *T*_*R*_(*t*_*i*_), where *T*_*R*_(*t*_*i*_) is the previously obtained raw estimate of the period for that moment in time *t*_*i*_. Only if their linear correlation coefficient is very close to unity (cf. Fig. 6(d)), purely periodic motion as estimated previously did indeed occur at *t*_*i*_. The resultant fine period estimates *T*_*F*_(*t*), on display in Fig. 6(e), provide more accurate estimations of the period and correctly identify intervals of pure periodicity. Moments without motion or with aperiodic motion are also correctly identified. The remaining difference between the true and the finely estimated results is at the beginning and end of intervals of pure periodicity – a phenomenon that is linked to the finite length of the second sliding window but is negligible for practical applications.

The results presented in Fig. 6 can also be interpreted in the following manner: consider Object 1 as a moving background (e.g. a fan) while we try to detect motion of Object 2 (e.g. a human). Although motion is detected all the time due to Object 1, the “smart” analysis of the temporal variations in the transmission changes enables the identification of when the detected motion ceases to be purely periodic due to additional motion of Object 2. This performance exemplifies the ability of the proposed sensor to operate in different and complex environments, without requiring many instructions from a user.

## Discussion

Imaging, tracking, and other sensing tasks (such as pose recognition of objects hidden behind thin scattering layers or around corners) are timely topics that have been explored with a variety of different approaches in optics on small scales^52–58^. Similar objectives on larger scales, corresponding to the microwave domain, are sought after in the quest for context-aware buildings. Transposing the ideas demonstrated with light passing through or reflected off thin multiply-scattering layers to disordered microwave cavities is not as trivial as invoking the theory of images. While wave transport is very sensitive to small perturbations in both problems, we are interested in detection *inside* rather than *behind* the complex medium which substantially complicates the problem.

Here, we have presented how any motion inside an indoor environment (a disordered cavity) can be detected, even outside the line-of-sight, working at a single frequency. We started off by exploring the benefit of using a dynamic metasurface aperture (DMA) that can emit different random radiation patterns and thereby couple differently to the cavity modes, enhancing the detection sensitivity in particular when working with intensity-only data. Then, we used the data to accurately identify intervals of purely periodic motion and to characterise their period, enabling “smart” motion detection. Periodicity is detectable for any irregularly shaped orbit in any orientation that the object follows over and over again. It is worth noting that in our analysis we only applied simple statistical tools to the transmitted signals to achieve the presented results. This processing simplification is not surprising since we believe our DMA-based approach enjoys a co-design of hardware and software for the task at hand. The DMA’s ability to probe the wave field in independent ways at rapid rates with a simple hardware distinguishes it from common hardware solutions, and satisfies the requirements of a sensor for complex environments (e.g. homes, vaults, etc.).

We believe that radiowave solutions, that do not require objects to collaborate, for instance, by being equipped with tags, have great potential for context-aware *Smart Buildings*. The presented results are a promising first step, and future work could envisage tasks such as counting the number of individuals in a room^9,22,59^, posture recognition by scattering cross-section estimation^23,60^, as well as object localisation given prior calibration by exploiting the fact that a disordered cavity naturally encodes the object position in Green’s function measurements^61–63^.
